# Mapping Global Potential Risk of Mango Sudden Decline Disease Caused by *Ceratocystis fimbriata*

**DOI:** 10.1371/journal.pone.0159450

**Published:** 2016-07-14

**Authors:** Tarcísio Visintin da Silva Galdino, Sunil Kumar, Leonardo S. S. Oliveira, Acelino C. Alfenas, Lisa G. Neven, Abdullah M. Al-Sadi, Marcelo C. Picanço

**Affiliations:** 1 Department of Plant Science, Universidade Federal de Viçosa, Viçosa, MG, Brazil; 2 Natural Resource Ecology Laboratory, Colorado State University, Fort Collins, CO, United States of America; 3 Department of Plant Pathology, Universidade Federal de Viçosa, Viçosa, MG, Brazil; 4 United States Department of Agriculture-Agriculture Research Service, Yakima Agricultural Research Laboratory, Wapato, WA, United States of America; 5 Department of Crop Sciences, Sultan Qaboos University, AlKhoud, Oman; 6 Department of Entomology, Universidade Federal de Viçosa, Viçosa, MG, Brazil; The University of Wisconsin - Madison, UNITED STATES

## Abstract

The Mango Sudden Decline (MSD), also referred to as Mango Wilt, is an important disease of mango in Brazil, Oman and Pakistan. This fungus is mainly disseminated by the mango bark beetle, *Hypocryphalus mangiferae* (Stebbing), by infected plant material, and the infested soils where it is able to survive for long periods. The best way to avoid losses due to MSD is to prevent its establishment in mango production areas. Our objectives in this study were to: (1) predict the global potential distribution of MSD, (2) identify the mango growing areas that are under potential risk of MSD establishment, and (3) identify climatic factors associated with MSD distribution. Occurrence records were collected from Brazil, Oman and Pakistan where the disease is currently known to occur in mango. We used the correlative maximum entropy based model (MaxEnt) algorithm to assess the global potential distribution of MSD. The MaxEnt model predicted suitable areas in countries where the disease does not already occur in mango, but where mango is grown. Among these areas are the largest mango producers in the world including India, China, Thailand, Indonesia, and Mexico. The mean annual temperature, precipitation of coldest quarter, precipitation seasonality, and precipitation of driest month variables contributed most to the potential distribution of MSD disease. The mango bark beetle vector is known to occur beyond the locations where MSD currently exists and where the model predicted suitable areas, thus showing a high likelihood for disease establishment in areas predicted by our model. Our study is the first to map the potential risk of MSD establishment on a global scale. This information can be used in designing strategies to prevent introduction and establishment of MSD disease, and in preparation of efficient pest risk assessments and monitoring programs.

## Introduction

**Fig 1:**
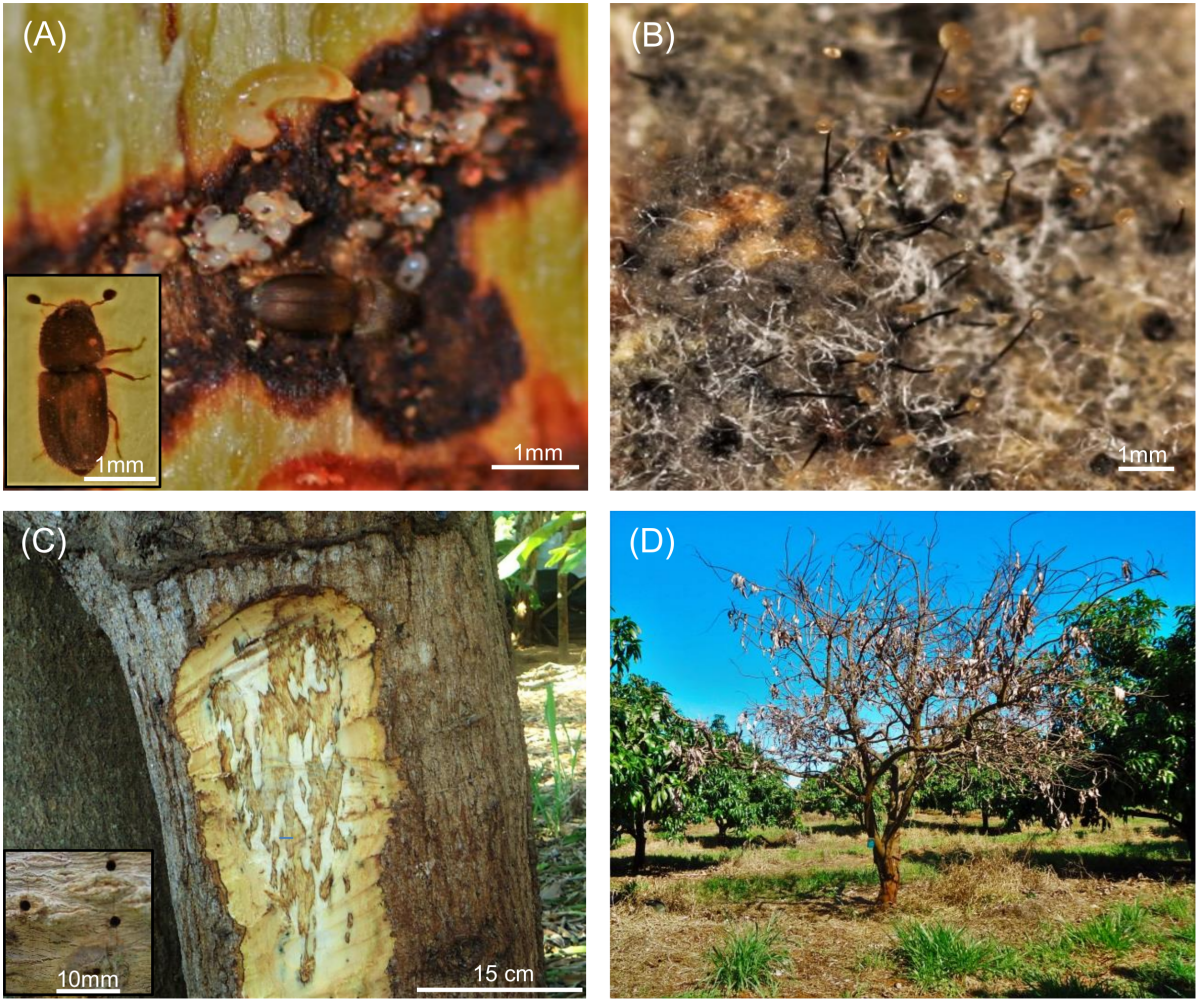
Mango Sudden Decline disease. (A) Eggs, larvae and adult female of the vector mango bark beetle, *Hypocryphalus mangiferae*, in an opened gallery. Inset shows an enlarged picture of the adult beetle, (B) Hyphae and perithecium with sticky ascospore masses of *Ceratocystis fimbriata*, (C) Section of a mango trunk showing the typical xylem discoloration caused by the fungal infection; entry and exit holes made by beetles on the surface of the bark (enlarged image in bottom left corner), and (D) A mango tree killed by Mango Sudden Decline disease.

Several species of fungi, including beneficial and harmful groups, can be found colonizing a single plant. Among these, approximately 8,000 fungal species are known to cause disease in plants, leading up to 100% loss of production [1]. *Ceratocystis fimbriata* sensu lato is described as complex of species considered the most important pathogens of woody plants, particularly in several plants of agronomic and forestry importance [2, 3]. *Ceratocystis* spp. can infect many different hosts such as mango, eucalyptus, sweet potato, coffee, cocoa and pomegranate [2–7]. The fungus *C*. *fimbriata* (Ellis and Halsted) sensu stricto is considered one of the most important species causing disease on mango (*Mangifera indica* L.) [3, 4, 6, 7]. The fungus *C*. *fimbriata* is the causal agent of the Mango Sudden Decline (MSD) disease, also referred to as Mango Wilt, an important disease that can lead to plant death in periods as short as two months after the initial infection (Fig 1) [2–7]. This species of *Ceratocystis* that causes the disease on mango was first reported in Brazil [6, 8]. The disease was later observed in Pakistan [9] and the Sultanate of Oman [6]. In these countries MSD has become one of the leading causes of mango crop losses [10–13]. In Oman, an estimated 60% of the production was lost in the fifth year after the introduction of MSD [14], killing over 200,000 mango trees, which resulted in the removal of 13% of the trees in order to prevent the spread of the disease [6]. In spite of the phytosanitary measures implemented by the Ministry of Agriculture and Fisheries in Oman, the spread of MSD disease continued [15]. In Pakistan, the losses varied between 20 and 60% of the production depending on which part of the country reported MSD losses [6, 12].

Different *Ceratocystis* species were identified as causing MSD in Brazil, Pakistan, and Oman. These include *C*. *manginecans* M. van Wyk, A. Adawi & M.J. Wingf., *C*. *mangivora* M. van Wyk & M.J. Wingf., and *C*. *mangicola* M. van Wyk & M.J. Wingf. The problem with the identification of these new *Ceratocystis* species was that they were based on unique internal transcribed spacer (ITS sequences) region of rDNA leading to wrong assignations of new species. A recent study with a detailed investigation showed that these species are all *C*. *fimbriata* (including those from Brazil, Oman and Pakistan), and indicated that these names are only synonyms of *C*. *fimbriata* [3]. To be consistent, these authors did not use only a single gene as the previous studies. They used three different sequencing genes (including ITS), morphological and sexual compatibility (interfertility) tests. They found that in mango only one species of *Ceratocystis* (*C*. *fimbriata*) is the causal agent of MSD.

The most important infection pathways of MSD are through infested soils and by vectors, the mango bark beetle *Hypocryphalus mangiferae* (Curculionidae: Scolytinae) (Fig 1A) [13, 15–17]. In the soil, the fungus produces aleurioconidias that work as structures of resistance that enable it to survive for long periods without the presence of a host [13, 18, 19]. The mango bark beetles have mycangia in elytra and mouthparts and are capable of carrying fungal structures over long distances [16]. Infestations in new areas usually begin in the branches of the trees where the beetles normally initiate attack [16]. Over time, the fungus may infect other parts of the plant, such as the trunk and roots, and afterwards, may remain in the soil [13, 15, 19]. Once the soil is infested with the fungus, it can result in the loss of an entire orchard, rendering the area unsuitable for mango cultivation. The best method to avoid losses due to MSD is to prevent its establishment in mango production areas. Prevention can be achieved by pruning and burning the branches or removing the whole tree immediately after the appearance of the first symptoms of the disease or attack by the beetles. This stops the progression of MSD establishment in the tree and prevents the fungus from infesting the soil [13, 20].

Understanding of the factors associated with the risk of establishment of MSD is urgently required by pest managers for management and prevention of this disease in mango production areas. Multiple factors can affect the establishment of pathogens in different locations in the world, including competition from other species, lack of host or dispersal vector, hostile climate, and natural barriers [21, 22]. Climate is one of the important abiotic factors that influences the global distribution of a species [22, 23]. Ecological niche models (ENMs) based on the quantitative relationship between environmental variables and species occurrences are used to predict areas of possible introduction, establishment, and spread of an invasive species [22, 24–27]. ENMs are based on classical concept of ‘‘niche” in ecology, and model potential or realized distribution of a species [28–29].

One type of ENMs are correlative models which are built by integrating species occurrences with spatial environmental variables of the study area [27, 30]. Correlative ENMs characterize the relationship between occurrence locations of a species with environmental characteristics of those locations, and use this to estimate the environmental suitability for a species in a specific location. Recent studies have demonstrated the predictive performance of these models [26, 27, 31–37]. Correlative models are widely used tools for assessing the risk of establishment of a variety of species including insects [25–27, 32, 33], aquatic organisms [23, 34], plants [37, 38], human diseases [39], vertebrates [30, 36, 40], and pathogens [41, 42]. The information on a species’ potential risk of establishment is helpful in developing a Pest Risk Assessment (PRA) (not developed for MSD yet), since the countries normally impose quarantine measures simply based on host species’ presence [43].

Despite the importance of maintaining an area free of MSD for mango cultivation, many countries apparently do not consider the risk of introduction of the disease because information on the potential risk of establishment of MSD in countries other than Brazil, Oman and Pakistan is lacking. With the availability of an ENM for MSD, existing phytosanitary restrictions may be re-evaluated and more attention given to the possibility of the introduction of the disease in other countries. Our objectives were to: (1) predict the global potential distribution of Mango Sudden Decline (MSD), (2) identify the mango growing areas that are under potential risk of MSD establishment, and (3) identify climatic factors associated with MSD distribution.

## Material and Methods

### Occurrence Data

**Fig 2:**
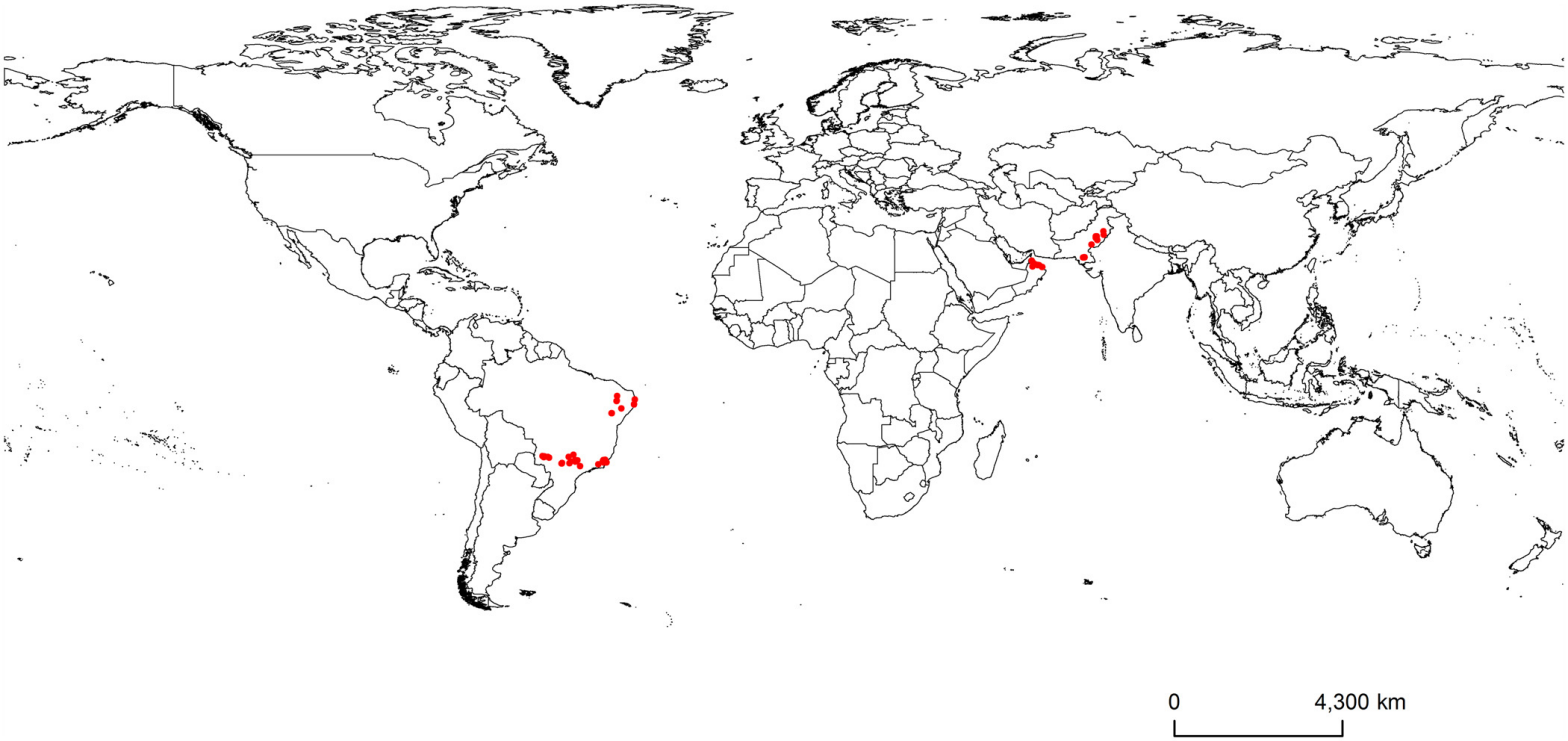
Global known occurrences of Mango Sudden Decline disease caused by *C*. *fimbriata* in mango trees.

We collected MSD occurrence data from all countries where the disease currently occurs: Brazil, Oman, and Pakistan [3]. MSD occurrence data points that cover all the regions inside these countries were collected (S1 Table). The data points for Brazil and Oman were collected in the field while conducting a study on phylogenetic analyses of *C*. *fimbriata* [3]. These data points correspond to locations with the presence of mango trees with symptoms of branch death, wilting foliage, bark discoloration, small holes in the bark, or sap exudation which indicates the presence of MSD disease [2, 3, 6, 7, 9]. At these locations, samples of the xylem showing discoloration (a characteristic of an infected tree; Fig 1) were collected from symptomatic mango trees in plantations, small farms, gardens, and along streets and roads for further confirmation of *C*. *fimbriata* presence. Samples were only taken at locations where the land owner had previously approved of sampling. No specific permissions were required for these countries since the species involved here are of agronomic interest and are not endangered or protected species. A total of 219 sites in Brazil, Oman, and Pakistan were confirmed for presence of the pathogen [3]. Since some of these sites were sampled more than once over the sampling period, we removed repeated occurrences corresponding to 80 unique points from Brazil and Oman. For Pakistan, the MSD disease presence data were collected from published papers that provided the coordinates of the locations of the diseased trees [44–46]. All taxonomic issues for the species were considered and only those that we were sure to be MSD caused by *C*. *fimbriata* or a synonym were considered [3]. Thus, a total of 94 unique occurrence records were collected from three countries where the disease is currently known to occur in mango trees (S1 Table, Fig 2) [6, 8, 9]. These records were reduced to 54 after applying spatial filtering using spThin, an R package (version 3.1.0) [47] to reduce spatial autocorrelation [48]. This method was chosen since it keeps the most locations possible and tends to perform better than other methods to reduce spatial autocorrelation [40]. The spThin checks for all possible combinations of filtered points using a minimum distance between them. From these new datasets, the one that keeps the largest number of records is selected to be used in the ENM [48]. Filtered occurrence data points were >10 km apart [40, 49]. This distance was used to ensure that each cell could have only one occurrence point since we used ~5-km spatial resolution climatic data in the model.

### Environmental Data

For this study a total of 20 environmental variables were considered including 11 variables derived from the monthly temperature, eight derived from the monthly precipitation, and the elevation above the sea level (Table 1). These variables were obtained from the WorldClim dataset (http://www.worldclim.org/) [50] at ~5-km spatial resolution. Data at ~5-km spatial resolution was used to account for potential spatial inaccuracies in species occurrence data, and climatic model accuracy due mainly to the low number of weather stations in some parts of the globe [22, 51]. These variables were derived using monthly temperature and precipitation data covering a period from 1950 to 2000, and represent average temperature, precipitation, seasonal variables, and climatic extreme indices [50]. These variables are considered biologically more meaningful than annual means of temperature and precipitation [50]. Only one variable from a group of highly correlated variables was included in the models (Pearson correlation coefficient, |r| ≥ 0.70) (S2 Table). The decision to include a variable was made based on its potential biological relevance to MSD distribution and ease of interpretation. For example, mean annual temperature was kept from a group of highly correlated variables since it is known to be very important for modeling different species distributions [26, 37, 39] and temperature is very important for MSD severity [2]. Thus, the final number of variables used for modelling MSD distribution was reduced to seven (Table 1 and S1 Table).

**Table 1:** Environmental variables considered in *C*. *fimbriata* niche models, and average percent contribution of environmental variables in the Mango Sudden Decline disease best MaxEnt model.

Variable	Percent contribution	Permutation importance	Min.	Max.	Mean	SD
**Mean annual temperature (bio1; °C)**	54.3	57.4	20.4	28.5	24.2	2.1
**Precipitation of coldest quarter (bio19; mm)**	13.7	4.1	9	821	116	154
**Precipitation seasonality (CV) (bio15)**	12.9	15.9	36	156	79	28
**Precipitation of driest month (bio14; mm)**	7.2	11.7	0	51	15	12
**Elevation (m)**	5.8	4.9	6	620	217	207
**Precipitation of wettest month (bio13; mm)**	4.2	5	20	319	164	90
**Mean diurnal range in temperature (bio2; °C)**	1.9	0.9	6.0	15.8	11.6	1.9
Isothermality (bio3)	-	-	39	74	57	11
Temperature seasonality (SD x 100) (bio4)	-	-	980	7917	3263	2028
Maximum temperature of warmest month (bio5; °C)	-	-	28.3	43.4	34.1	4.6
Minimum temperature of coldest month (bio6; °C)	-	-	4.5	20.9	12.8	3.6
Temperature annual range (bio7; °C)	-	-	8.9	38.5	21.3	6.9
Mean temperature of wettest quarter (bio8; °C)	-	-	20.2	34.4	25.6	3.4
Mean temperature of driest quarter (bio9; °C)	-	-	17	33.5	22.5	4.5
Mean temperature of warmest quarter (bio10; °C)	-	-	22.9	34.8	27.9	3.9
Mean temperature of coldest quarter (bio11; °C)	-	-	13.5	26.2	19.6	2.3
Mean annual precipitation (bio12; mm)	-	-	73	2093	893	565
Precipitation of wettest quarter (bio16; mm)	-	-	49	914	429	254
Precipitation of driest quarter (bio17; mm)	-	-	0	178	61	46
Precipitation of warmest quarter (bio18; mm)	-	-	0	685	342	232

Values were averaged across 10 replicate runs. General statistics were calculated using all occurrences (n = 94). Min. is minimum, Max. is maximum, and SD is standard deviation.

Note: Bold font indicates variables selected for model building. Source of data: WorldClim (http://www.worldclim.org/bioclim) [50].

### Model Development and Validation

The correlative maximum entropy based model or MaxEnt algorithm (version 3.3.3k) [52] was used to assess the global potential distribution of MSD. MaxEnt is a machine learning method and estimates the probability distribution of the maximum entropy for a species constrained by the sample data and it is based on multiple environmental variables using a high-dimensional dataset [21, 22, 24–27, 52]. MaxEnt was chosen because it uses species presence and background data (absence data are not needed) and also works well with small sample sizes [35, 53]. MaxEnt estimates the environmental suitability for a species based on presence records and randomly generated background points by finding the maximum entropy distribution and its geographical projection [52]. It produces an index of suitability that varies from 0 (unsuitable) to 1 (most suitable) [25, 26, 52]. A total of 50,000 background points were randomly selected from areas where *C*. *fimbriata* currently occurs. This number was chosen since it is more appropriate when working at a global scale [54, 55]. A sampling bias was suspected because the data were collected near roads and more accessible areas and from sources where we could not control the sampling process. Thus, a bias surface using a kernel density estimate was generated using the SDMToolbox [54]. The bias surface will result in a raster where cells with lower values will represent places with lower bias [52]. The bias surface was used to account for sampling intensity and potential sampling bias [55].

Different settings in MaxEnt were adjusted to find an optimal model for MSD disease potential distribution since default settings are not always the best [27, 55, 56]. These adjustments consisted of different combinations of regularization multiplier (RM) and feature types generating many different models. The RM controls the number of parameters and consequently the model complexity [56, 57]. The RM values used were 1.0, 1.5, and 2.0. An RM value <1 generates models that are very restricted (not desired for world predictions) and values >1 would result in simpler models with a broader potential distribution [52]. These values were used in combination with different sets of MaxEnt features (i.e. linear [L], quadratic [Q], product [P], threshold [T], and hinge [H]). The ‘fade-by-clamping’ option was used to prevent extrapolations outside the environmental range of the training data [58]. The percent contribution, permutation importance, and ‘Jackknife’ (leave-one-out) technique in MaxEnt [52] were used to estimate the predictive power of different environmental predictors. The percent contribution estimates the contribution of a variable to the model and the permutation importance indicates how much the model depends on that variable. 'Jackknife' procedure was used in MaxEnt to account for the importance of a variable over 10-fold-cross-validation. This is done by evaluating different models in two situations: using only the variable by itself and using all other variables excluding that one in question. The results are the training gain and the area under the curve (AUC) for each environmental variable for each situation. The MaxEnt generated response curves that were used to show the relationships between predicted probabilities of presence of the disease with respect to the variation within each environmental variable. These curves were analyzed and models showing complex curves (highly irregular shape) were not considered for further evaluations; models that included predictors with these erratic curves are not used because they are considered biologically unrealistic. We considered complex curves as those with the highly jagged or multimodal behavior which normally does not happen with species’ responses to environmental variables. Only thirteen models were considered for further evaluations.

The evaluation metrics for ranking the models’ performance were the AUC_cv_ (area under the receiver operating characteristic [ROC] curve) [59] and the test sensitivity (i.e., percentage of correctly predicted presences) at 0% and 10% training Omission Rates (OR) [33, 60]. OR was used in addition to AUC_cv_ because AUC_cv_ alone is not the best approach to choose between different models when working with the prediction of invasive potential of a species. The problem with AUC_cv_ is that it gives the same weight for sensitivity and specificity, while in case of prediction of invasive potential of a species, sensitivity should receive more attention [61–62]. Test sensitivity thresholds at 0% and 10% means that zero and ten percent, respectively, of training presence locations for MSD fall outside the predicted suitable area. For that we ran a 10-fold cross-validation in MaxEnt to calculate AUC_cv_ and OR. The AUC_cv_ measures the ability of the model to discriminate presence from background. AUC_cv_ value of 0.5 shows that model predictions are not better than random; values below 0.5 are worse than random; between 0.5–0.7 indicate poor performance; between 0.7–0.9, reasonable or moderate performance; and values higher than 0.9 indicates high performance [63]. For the OR, the expected value of test omission rate at 0% training OR is 0, whereas at 10% training OR threshold it is 0.10; higher than expected ORs show poor performance of the models [40]. The best models were ranked based on 10% training OR, 0% training OR, and AUC_cv_, respectively [26, 56, 60].

To identify the mango growing areas that are under potential risk of MSD establishment mango yield data were obtained from the Earth Stat (http://www.earthstat.org/) [64] with 10x10 km resolution. These data represent the average yield of mango in tons per hectare for the period from 1997–2003. These data were reclassified to a binary map using Reclassify tool in ArcGIS, version 10.2 (ESRI, Redlands, CA). Cells with zero values and no data values were converted to zero, and cells with all other values were converted to one, thus generating a map with zero representing cells with no mango production and one for those areas where mango is produced. This binary layer of mango production reports using the Expand tool in ArcGIS was used to reduce problems due to the fact that in some areas the reports were just single cells, they were difficult to visualize, and the data for some regions were of low accuracy [64]. Finally, to estimate the suitability for the disease only in mango production areas, the MaxEnt predicted output (the output of the model) was extracted to mango production areas. The extended binary map of mango production was multiplied by the MaxEnt predicted output, to keep the suitability for MSD disease (in relation to the model) in cells with mango production reports and converted areas with no mango production to zero.

## Results

As per observed occurrences, MSD disease occurs in areas with mean annual temperature between 20.4–28.5°C, mean annual precipitation between 73–2093mm, and below 620m of elevation (Table 1). The mean annual temperature (54.3%), precipitation of coldest quarter (13.7%), precipitation seasonality (12.9%), and precipitation of driest month (7.2%) contributed most to MSD disease potential distribution (Table 1). The mean annual temperature also showed the highest permutation importance (57.4%), followed by precipitation seasonality (15.9%), precipitation of the driest month (11.7), and precipitation of the wettest month (5%).

All 13 MaxEnt models evaluated to determine MSD disease potential distribution performed better than random with test AUC_cv_ values higher than 0.5 (Table 2). Average AUC_cv_ values based on 10-fold cross validation varied from 0.939–0.974 (Table 2). These models also had low test omission rates with values at 0% training OR varying from 0.017–0.093 (expected value is 0), and at 10% training OR from 0.110–0.223 (expected value is 0.10); values higher than the expected ORs show poor performance of the models (Table 2). The best model included seven environmental variables, Linear, Quadratic, and Hinge (LQH) features, regularization multiplier = 1.5, and had the lowest test OR, at 10% and 0% respectively (Table 2).

**Table 2:** Summary of performance statistics of *C*. *fimbriata* MaxEnt models.

Model No.	Variables	MaxEnt settings		Test AUC_cv_ (±SD)	Test OR		Model Rank^a^
		Features	RM		0%	10%	
**1**	**bio1, bio2, bio13, bio14, bio15, bio19, Elevation**	**LQH**	**1.5**	**0.970 ± 0.012**	**0.017**	**0.110**	**1**
2	Same as above	LQH	2.0	0.969 ± 0.011	0.037	0.133	6
3	Same as above	LQPTH	1.5	0.971 ± 0.012	0.093	0.150	10
4	Same as above	LQPTH	2.0	0.965 ± 0.014	0.090	0.200	11
5	Same as above	LQP	1.0	0.951 ± 0.017	0.057	0.130	4
6	Same as above	LQP	1.5	0.946 ± 0.017	0.033	0.143	8
7	Same as above	LQP	2.0	0.939 ± 0.021	0.020	0.110	2
8	Same as above	LH	1.5	0.971 ± 0.012	0.020	0.133	5
9	Same as above	LH	2.0	0.967 ± 0.013	0.033	0.150	9
10	Same as above	LQPT	1.5	0.956 ± 0.021	0.057	0.223	13
11	Same as above	LQPT	2.0	0.948 ± 0.020	0.040	0.220	12
12	Same as above	LQPH	1.5	0.974 ± 0.010	0.070	0.137	7
13	Same as above	LQPH	2.0	0.967 ± 0.012	0.053	0.110	3

See Table 1 for variables’ full names. L, Q, P, T and H are linear, quadratic, product, threshold and hinge features, respectively. RM is regularization multiplier, and SD is standard deviation. OR is test omission rate. Test AUC_cv_ is MaxEnt 10-fold cross-validation Area Under the ROC curve.

^a^The model with the highest performance is highlighted in bold.

**Fig 3:**
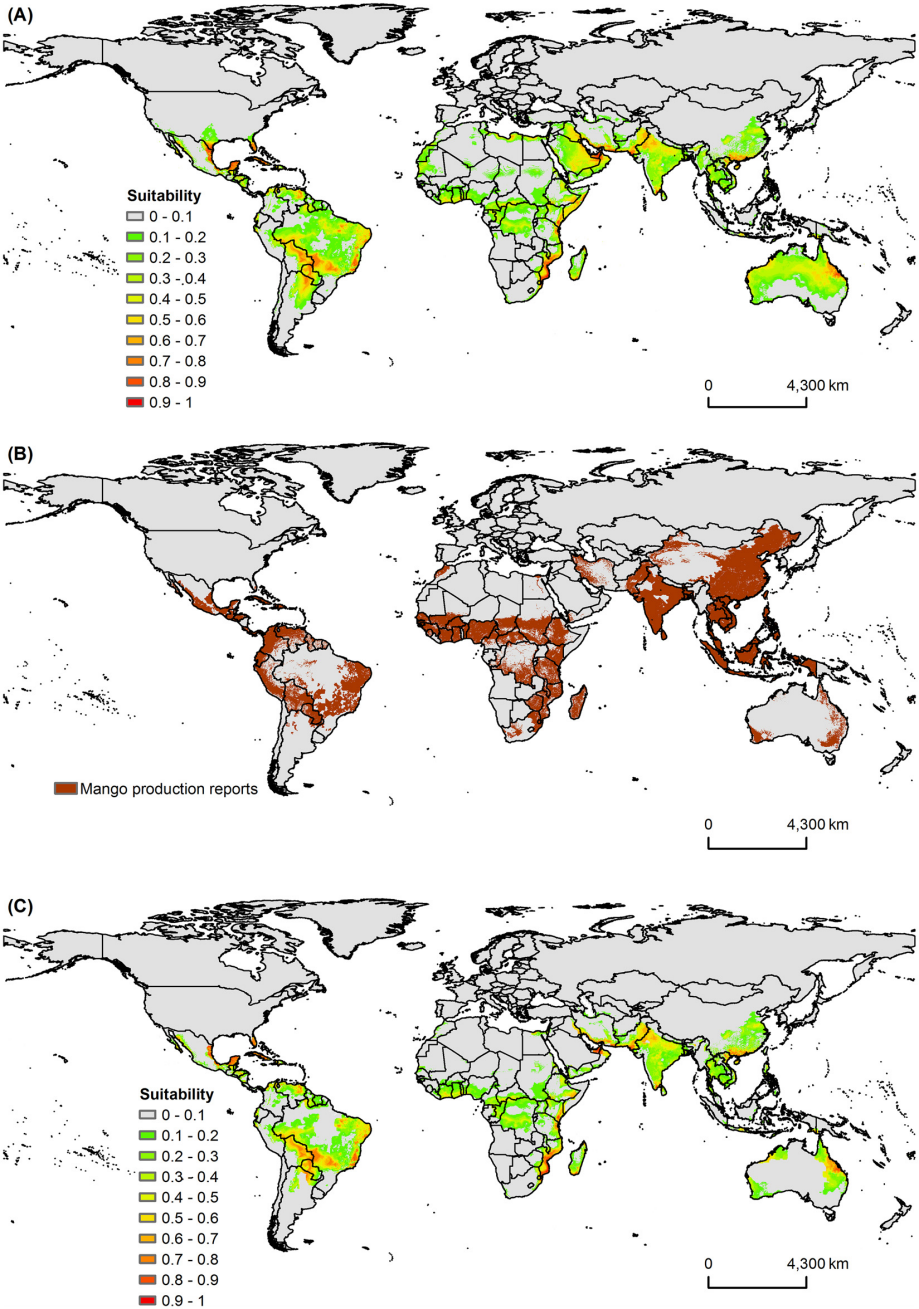
Global Potential Risk of Mango Sudden Decline Disease. Maps of (A) global potential distribution of *C*. *fimbriata* using MaxEnt model, (B) global mango growing areas (source of data: Earth Stat, http://www.earthstat.org/; used with permission of Peder Engstrom from EarthStat.org/U Minnesota under a CC BY license, original copyright [2008] [64]), and (C) potential distribution of *C*. *fimbriata* in mango growing areas.

Predictions of the best MaxEnt model for MSD disease covered all of its current known occurrences (Figs 2 and 3A). The model predicted highly suitable areas in South America, southern North America, Central America, parts of Africa, northern Australia, Middle Eastern countries (e.g. Oman, Saudi Arabia, and United Arab Emirates) and parts of Asia (Fig 3, and S1–S4 Figs). This also includes countries such as Brazil, Oman, and Pakistan, where the disease already occurs in mango (Fig 3A, S1 and S4 Figs). Mango is grown in many countries in the world, primarily those in tropical areas and some subtropical areas (Fig 3B). Almost all of these mango growing areas are suitable for MSD disease establishment except for few areas in South Africa, Colombia, Ecuador, northeastern parts of China, northern Pakistan, and northern and northeastern parts, and Western Ghats of India (Fig 3C and S4 Fig).

**Fig 4:**
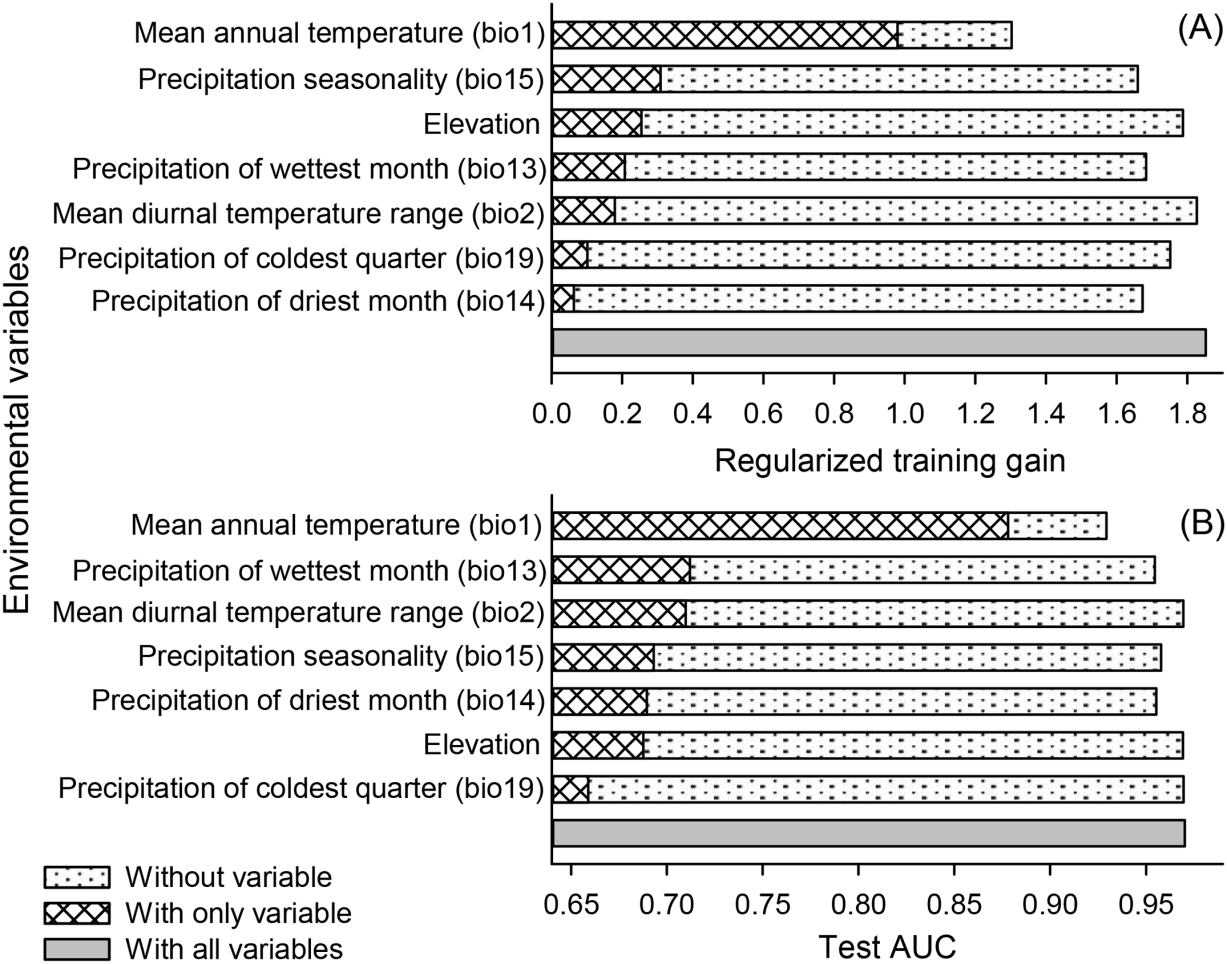
Relative importance of the environmental variables based on the Jackknife test. The figures show each variable’s contribution to (A) regularized training gain, and (B) test AUC in *C*. *fimbriata* model.

**Fig 5:**
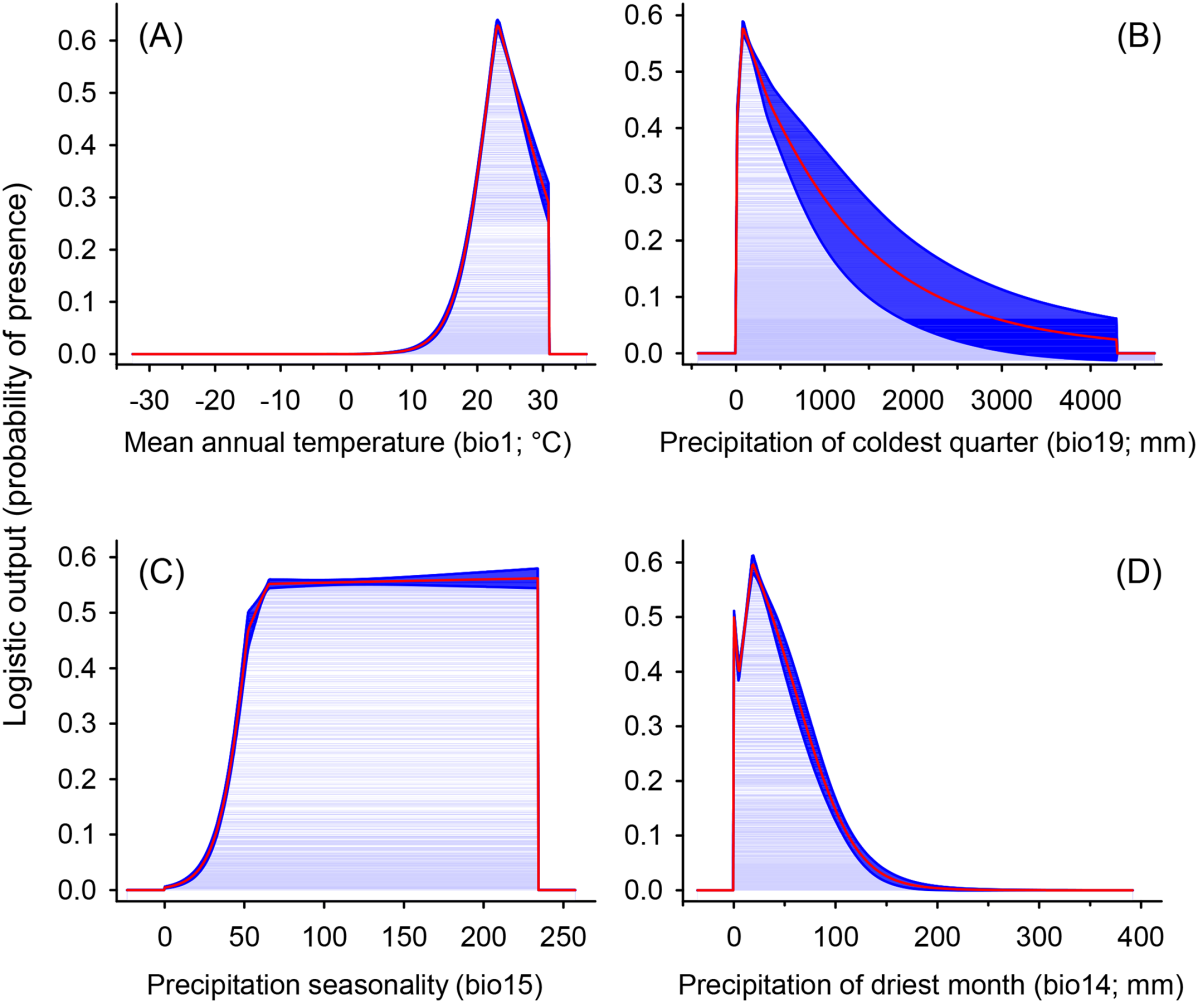
Response curves of the best predictors of *C*. *fimbriata* in the best model. (A) mean annual temperature (bio1; °C), (B) precipitation of coldest quarter (bio19; mm), (C) precipitation seasonality (Coefficient of variation; bio15), and (D) precipitation of driest month (bio14; mm). Red lines are the mean response curves and blue margins are ± Standard Deviation calculated over 10 replicates.

The ‘Jackknife’ test of variable importance showed that the mean annual temperature had the most information that was not present in other variables contributing most to the model, with the highest regularized training gain and AUC (Fig 4A and 4B). The relationships between predicted probabilities of presence of the disease with respect to the variation within each environmental variable are presented in Fig 5. The highest suitability for MSD disease presence is in areas with mean annual temperatures around 23°C, with the suitability decreasing sharply with the increasing or decreasing mean annual temperature, with no predictions of occurrence in temperatures below 10°C or above 30°C (Fig 5A). The suitability was higher in areas with low precipitation of coldest quarter (<1000 mm), decreasing with the increase in precipitation of coldest quarter (Fig 5B). The suitability for MSD disease presence was low in areas with low precipitation seasonality (<50), with the suitability increasing exponentially in areas where the precipitation seasonality is higher than 25, until reaching a plateau at 50 (Fig 5C). The suitability for MSD presence was also higher in areas of low precipitation and zero in areas with precipitation over 150mm during the driest month (Fig 5D).

## Discussion

This is the first study to map Mango Sudden Decline disease potential distribution on a global scale. The cross-validation procedure indicated that all of the models performed much better than would be expected at random and had a high validation statistic (test AUC >0.9; Table 2; Fig 4). The potential distribution of the disease was closely related to its current known occurrences in mango plants in Brazil, Oman, and Pakistan (Figs 2 and 3A, S1 and S4 Figs). The MaxEnt model predicted suitable areas in countries where the disease does not already occur in mango, but where mango is grown (Fig 3C, S1–S4 Figs). Among these areas are the largest mango producers in the world including India, China, Thailand, Indonesia, Mexico, Pakistan, and Brazil, which together correspond to more than 85% of the world’s mango production [65]. In addition, the model also predicted areas of high susceptibility beyond the current occurrence within the countries where the disease currently occurs; for example, Brazil, Oman, and Pakistan (S1 and S4 Figs).

The occurrence of hosts and vectors is important for the establishment of a pathogen and may in some cases be one of the causes of failure in the colonization of new areas [22, 66]. The MSD disease vector, the mango bark beetle *H*. *mangiferae*, was present at all locations used in the MaxEnt model. Therefore, places predicted as susceptible to the occurrence of the disease may potentially have the occurrence of its vector, the mango bark beetle. This beetle is also known to occur beyond the location data we collected, including Florida, Mexico, Venezuela, Australia, and India [15, 67]. All of these sites were observed in our study as being susceptible to the establishment of MSD. However, these occurrences are of very low spatial accuracy and it was not possible to model the beetles. As host and vector are very important to the establishment of the disease, this fate increases the likelihood that the disease may establish in areas predicted by our model; specifically, areas where the vector and the host already occur or may occur (Figs 2 and 3).

Mean annual temperature was one of the most important variables associated with the distribution of the MSD disease (Table 1). Several studies have shown that mean annual temperature is among the factors that contributes most to species distribution mainly when working at a global scale [33, 37, 39]. The model predicted higher suitability for MSD in locations where temperatures average 23°C, with a significant decrease in suitability with a decrease or increase in the mean annual temperature. Studies on *C*. *fimbriata* demonstrated that the optimum temperature for sporulation of the fungus under laboratory conditions is between 24 and 26°C, very close to the values estimated by our model (Fig 5A) [2, 68]. The difference between the values estimated by our model and under laboratory conditions are probably due to the fact that the model uses a series of 50 years of climatic data, and in laboratory the temperature is always the same to evaluate the sporulation. The suitability was higher at lower elevations. Ecological niche modeling studies with other species demonstrate that the elevation has great influence on species distributions [33, 69]. This may be explained by the relationship of elevation with humidity and temperature variations [31], which influences *C*. *fimbriata* sporulation [12]. However, MSD disease was observed in a wider range of precipitation levels (73–2093mm). The occurrence of the disease in low rainfall sites is only possible due to irrigation in these areas (e.g., Oman and Pakistan), which makes development of the host and vector possible and in turn, the disease. Furthermore, it was observed that the disease is more likely to occur in areas with well-defined dry and rainy seasons, since it is more likely to occur in areas with a high coefficient of variation in precipitation (i.e., precipitation seasonality; Fig 4C). It is possible due to the fact that dry season makes the host more susceptible to the fungus and rainy season offers better conditions to the sporulation of *C*. *fimbriata* [2, 12, 68].

The climatic conditions found in this study for the disease are very close to ideal conditions for the mango tree development. The mango tree has optimum range of temperature ranging from 24 to 30°C, better development at elevations <600m, poor development in regions with high rainfall (>2000mm), and small differences in precipitation between dry and rainy seasons [10]. These factors indicate that the fungus has climatic requirements very similar to the host. This resulted in the model predictions for the occurrence of the MSD disease in almost all locations where mango is cultivated, which reinforces the robustness of the model (Fig 3, S1–S4 Figs).

*Ceratocystis fimbriata* is a soil-borne pathogen which can live in the soil for long periods of time, thus making it almost impossible to eradicate in infected areas [13, 18, 19]. We identified many areas across the globe that have suitable climatic conditions for the establishment of MSD disease. These results can be used in Pest Risk Assessments (PRA) program by National Plant Protection Organizations (NPPOs). It can be done by including MSD in the list of risk diseases and monitoring unintentional introductions of *C*. *fimbriata*. Some pathways analyses show that introductions in new areas may occur through contaminated soil, infected tissue or even by the vector *H*. *mangiferae* which may carry some structures of the fungus in its mouthparts and digestive tract [13, 15–17]. Other studies have demonstrated that other bark beetle species, such as *Xyleborus affinis*, may also be involved in the spread of the fungus, but does not seem to be as much important as *H*. *mangiferae* [16, 70]. Thus, the training of farmers to identify symptoms of the disease in trees and the mango bark beetle vector are advisable before the disease enters the country. This can reduce the initial propagule pressure and thus make it easier to prevent its establishment in the country. It would be much worse if it reaches higher levels of infections or infests the soil [13, 18, 19, 71, 72].

The results of this study should be interpreted with caution. Correlative niche models like MaxEnt may have prediction uncertainties [55]. These uncertainties are primarily due to the quality of occurrence data, sampling bias, resolution of spatial data layers, species characteristics, and spatial autocorrelation [21, 55, 73–75]. The MaxEnt model has a very user friendly interface which makes the generation of results somewhat easier. However, several adjustments can be made, which can have a great influence on the model and consequently on its accuracy [26, 32, 33, 55]. These adjustments include selection of background points and extent, value of regularization multiplier, and selection of feature types [52]. Considering these potential pitfalls in the modeling process, we took utmost care in model calibration and thus generating predictive models that are consistent with the current known distribution of the species (Figs 2 and 3). This can be observed in the quality of response curves (no highly jagged or multimodal response observed) and good validation results (Table 2; Figs 4 and 5). A number of modeling algorithms are available to model the habitat suitability for a species. All these methods will generate different predictions which can also lead to uncertainties. Some authors advised to use different techniques and thus preventing this type of uncertainty [55]. Here we only used MaxEnt algorithm because it seems to be more appropriate to our situation (i.e., it uses species presence and background data and also works well with small sample sizes) [35, 53]. However, other modelling methods can have different predictions from our model.

This study provides important information on the potential risk of establishment of MSD disease using a MaxEnt model. These results can be used in designing strategies to prevent introduction and establishment of MSD disease, and in preparation of efficient Pest Risk Assessment and monitoring programs by countries where MSD disease currently occurs and in other countries that are at risk. Efforts can be made by these countries for effective monitoring and surveillance of potential introduction of this disease via trade from currently infested countries (Brazil, Oman, and Pakistan). Countries like India and Venezuela, that produce mangoes and are near countries where the disease is already established, should devote more time and efforts in preventing MSD introduction. This is worse for these countries because the vector *H*. *mangiferae* already occurs there (showing that they offer suitable conditions for the beetles), and the possibility of migration from infested countries is higher compared to the ones located distant from infested countries (natural dispersion). Also, since the beetle is small it may enter in vehicles and other transported materials without being noted (human mediated dispersion). So efforts on monitoring the disease in suitable places areas are required.

## Supporting Information

S1 Fig Enlarged maps. (A) potential distribution using MaxEnt model, and (B) potential distribution in mango growing areas of *C*. *fimbriata* in South America.
(TIF)

S2 Fig Enlarged maps. (A) potential distribution using MaxEnt model, and (B) potential distribution in mango growing areas of *C*. *fimbriata* in the biggest mango producers in North America.
(TIF)

S3 Fig Enlarged maps. (A) potential distribution using MaxEnt model, and (B) potential distribution in mango growing areas of *C*. *fimbriata* in China, Indonesia, Philippines Thailand, and Taiwan.
(TIF)

S4 Fig Enlarged maps. (A) potential distribution using MaxEnt model, and (B) potential distribution in mango growing areas of *C*. *fimbriata* in Pakistan, Oman, India, Bangladesh, and Sri Lanka.
(TIF)

S1 Table Country, location, species, and coordinate points (latitude and longitude) of the 94 occurrence records used in the model. (DOCX)

S2 Table Cross-correlation (Pearson correlation coefficient, r) among environmental variables. (DOCX)
