# Supplementary figures and images for: Mapping Global Potential Risk of Mango Sudden Decline Disease Caused by Ceratocystis fimbriata
Source: PLoS One. 2016 Jul 14;11(7):e0159450. doi: 10.1371/journal.pone.0159450 (PMC4944967; doi:10.1371/journal.pone.0159450)

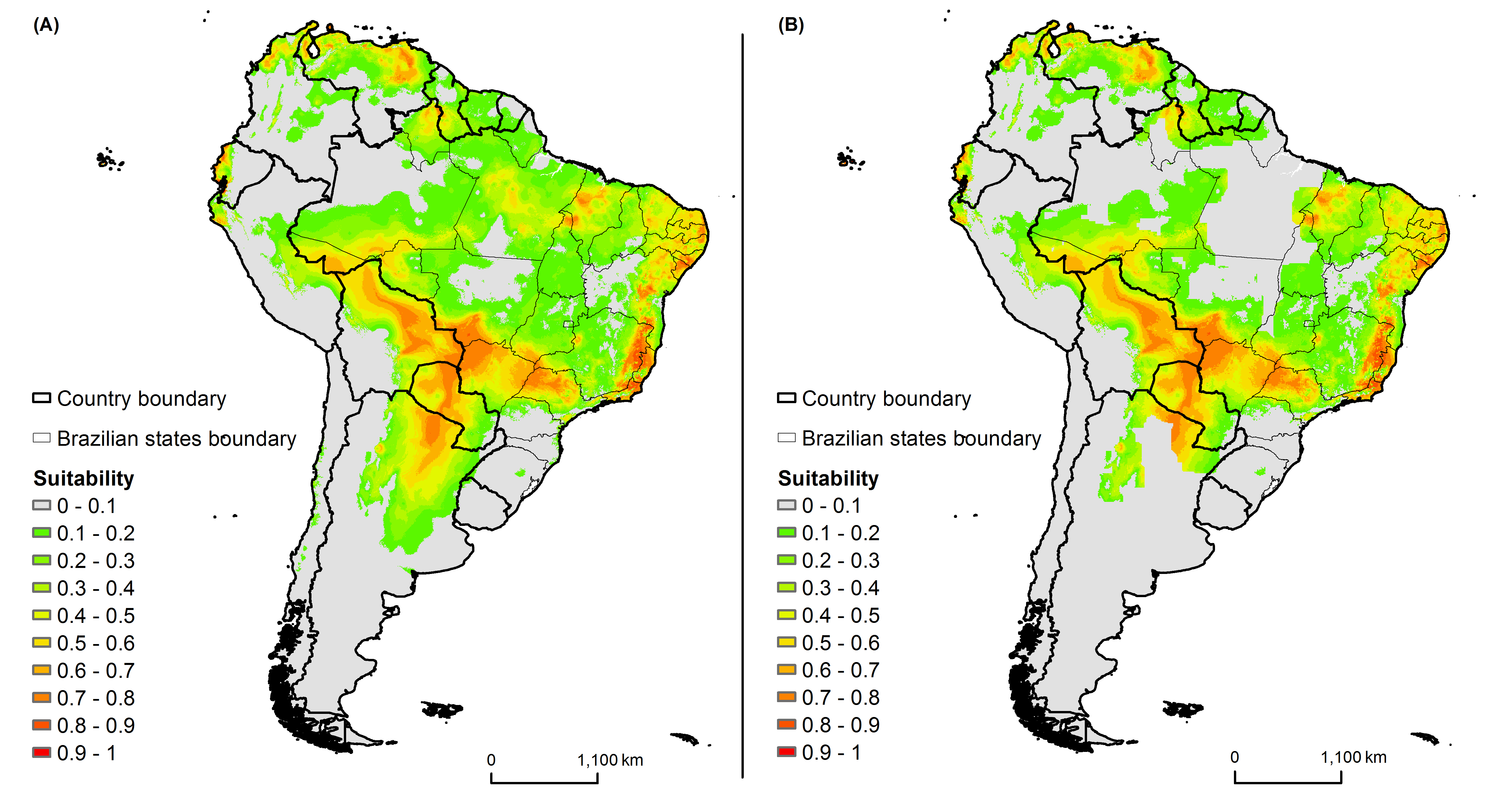

Supplement: S1 Fig — (A) potential distribution using MaxEnt model, and (B) potential distribution in mango growing areas of C. fimbriata in South America. (TIF) [file pone.0159450.s001.tif]

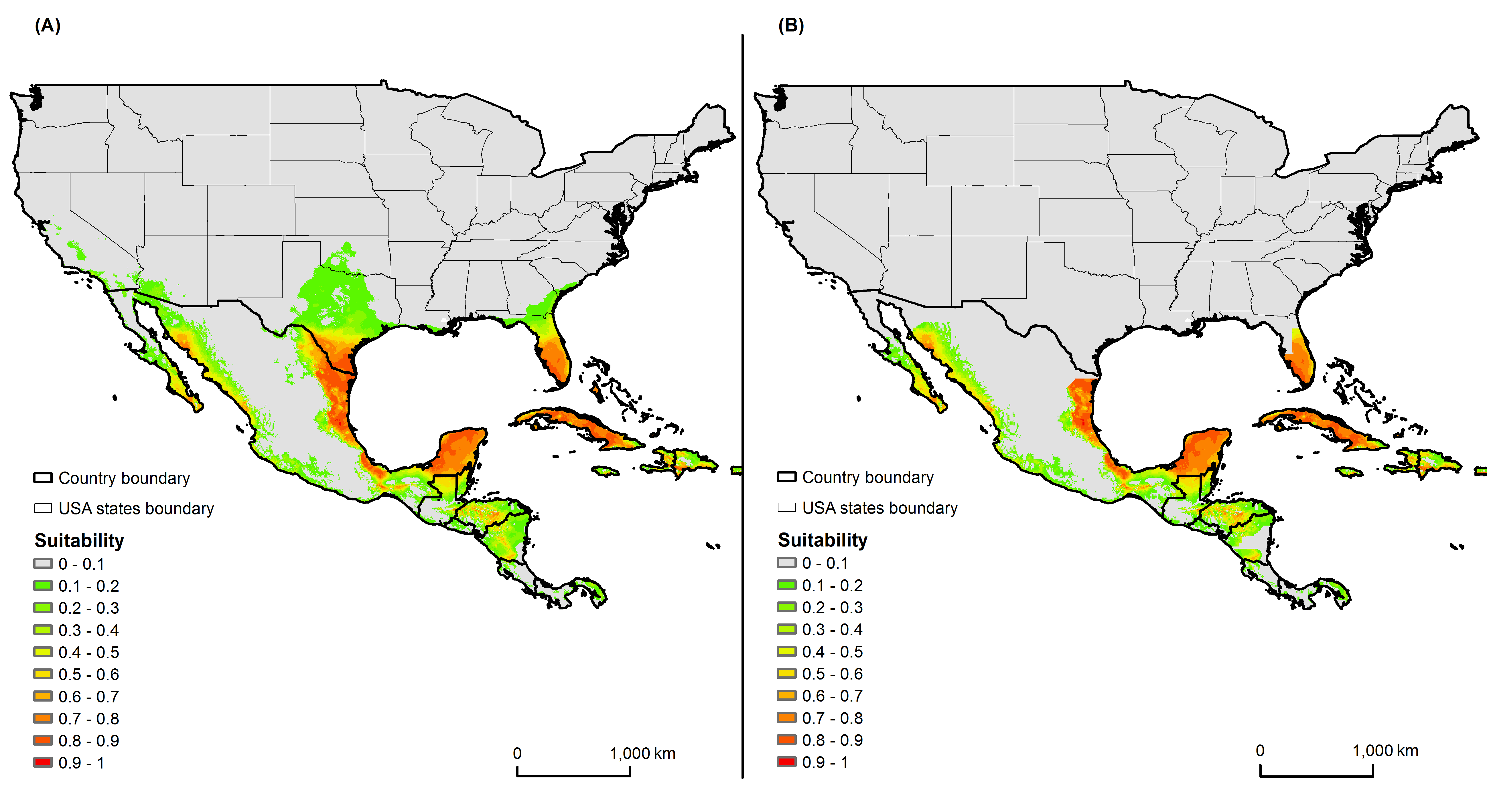

Supplement: S2 Fig — (A) potential distribution using MaxEnt model, and (B) potential distribution in mango growing areas of C. fimbriata in the biggest mango producers in North America. (TIF) [file pone.0159450.s002.tif]

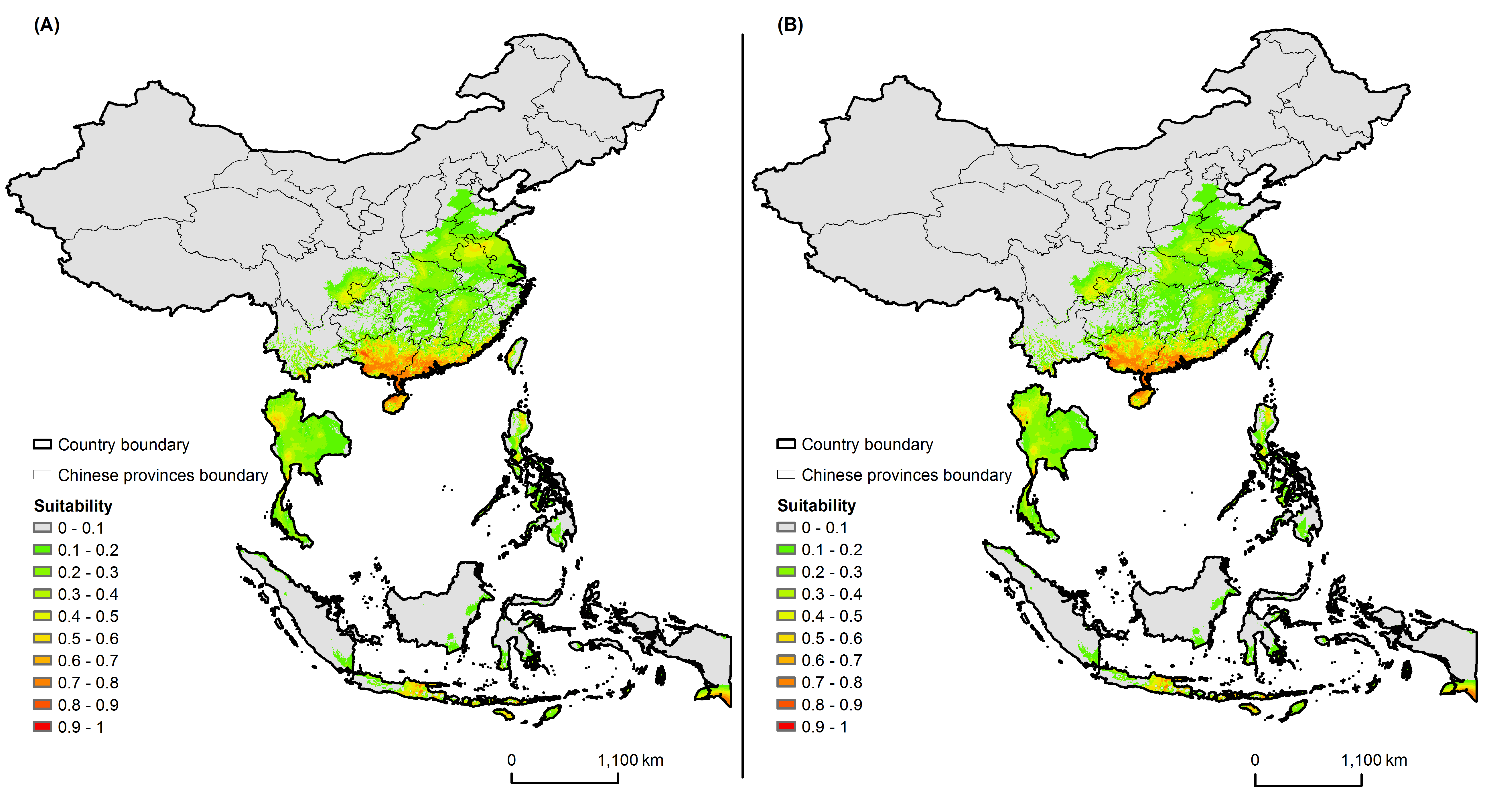

Supplement: S3 Fig — (A) potential distribution using MaxEnt model, and (B) potential distribution in mango growing areas of C. fimbriata in China, Indonesia, Philippines Thailand, and Taiwan. (TIF) [file pone.0159450.s003.tif]

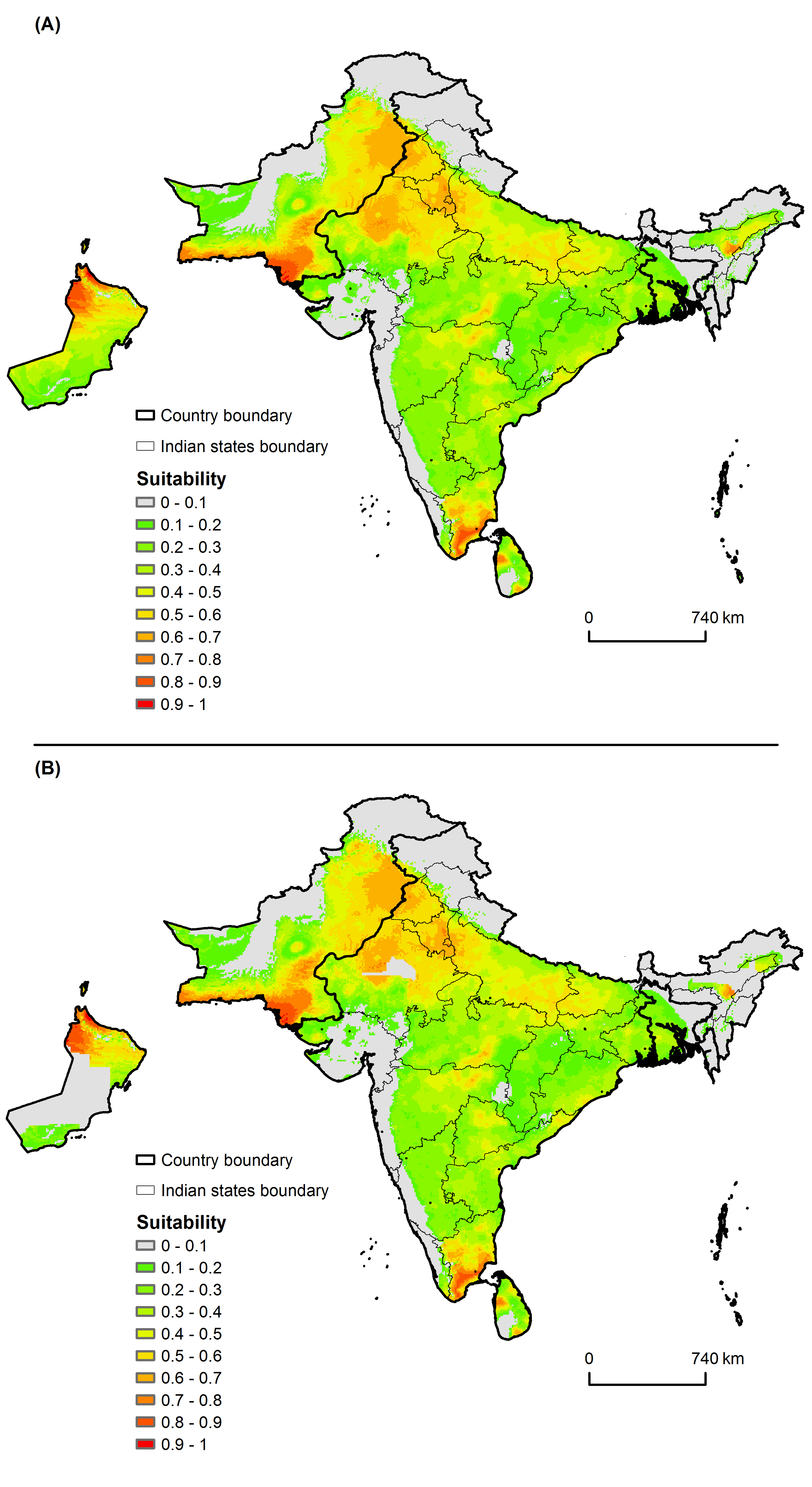

Supplement: S4 Fig — (A) potential distribution using MaxEnt model, and (B) potential distribution in mango growing areas of C. fimbriata in Pakistan, Oman, India, Bangladesh, and Sri Lanka. (TIF) [file pone.0159450.s004.tif]
