# Supplementary material for: Mapping Global Potential Risk of Mango Sudden Decline Disease Caused by Ceratocystis fimbriata
Source: PLoS One. 2016 Jul 14;11(7):e0159450. doi: 10.1371/journal.pone.0159450 (PMC4944967; doi:10.1371/journal.pone.0159450)
Supplement: S1 Table — (DOCX) [file pone.0159450.s005.docx]

**S1 Table. Country, location, species, and coordinate points (latitude and longitude) of the 94 occurrence records used in the model.**

| **Country** | **Location name/State** | **Species** | **Latitude** | **Longitude** |
| --- | --- | --- | --- | --- |
| Oman | Barka | Ceratocystis fimbriata | 23.65234 | 57.86925 |
| Oman | Quriyat (Daghmar Al-Blad) | Ceratocystis fimbriata | 23.19403 | 58.98274 |
| Oman | Quriyat (Hail Al-Ghaf) | Ceratocystis fimbriata | 23.17309 | 58.92360 |
| Oman | Al-Seeb (Al-Hail South) | Ceratocystis fimbriata | 23.61697 | 58.22511 |
| Oman | Barka | Ceratocystis fimbriata | 23.64731 | 57.88075 |
| Oman | Barka | Ceratocystis fimbriata | 23.66272 | 57.88663 |
| Oman | Swaiq (Bateha Hilal) | Ceratocystis fimbriata | 23.78582 | 57.38114 |
| Oman | Swaiq | Ceratocystis fimbriata | 23.82009 | 57.38289 |
| Oman | Swaiq (Al-Shater) | Ceratocystis fimbriata | 23.81510 | 57.37584 |
| Oman | Sohar (Al-Waqaibah) | Ceratocystis fimbriata | 23.33749 | 56.72079 |
| Oman | Sohar (Al-Tarif) | Ceratocystis fimbriata | 24.34303 | 56.71808 |
| Oman | Sohar (Al-Tarif) | Ceratocystis fimbriata | 24.36673 | 56.70150 |
| Oman | Shinas | Ceratocystis fimbriata | 24.62906 | 56.51456 |
| Oman | Saham | Ceratocystis fimbriata | 24.15152 | 56.85990 |
| Oman | Al-Koudh | Ceratocystis fimbriata | 23.59849 | 58.16498 |
| Brazil | Minas Gerais | Ceratocystis fimbriata | -19.94778 | -48.96556 |
| Brazil | Rio de Janeiro | Ceratocystis fimbriata | -22.14267 | -43.26350 |
| Brazil | Rio de Janeiro | Ceratocystis fimbriata | -21.35611 | -41.70889 |
| Brazil | Rio de Janeiro | Ceratocystis fimbriata | -21.71611 | -41.34139 |
| Brazil | Rio de Janeiro | Ceratocystis fimbriata | -21.62778 | -41.61111 |
| Brazil | Rio de Janeiro | Ceratocystis fimbriata | -21.62917 | -41.62667 |
| Brazil | Rio de Janeiro | Ceratocystis fimbriata | -21.65278 | -41.72222 |
| Brazil | Rio de Janeiro | Ceratocystis fimbriata | -21.68750 | -41.79806 |
| Brazil | Rio de Janeiro | Ceratocystis fimbriata | -21.63389 | -41.72333 |
| Brazil | Rio de Janeiro | Ceratocystis fimbriata | -21.63667 | -41.76389 |
| Brazil | Rio de Janeiro | Ceratocystis fimbriata | -21.66639 | -41.84111 |
| Brazil | Rio de Janeiro | Ceratocystis fimbriata | -21.69694 | -42.03500 |
| Brazil | Rio de Janeiro | Ceratocystis fimbriata | -21.67667 | -42.07028 |
| Brazil | Rio de Janeiro | Ceratocystis fimbriata | -21.53944 | -42.18028 |
| Brazil | Rio de Janeiro | Ceratocystis fimbriata | -21.17917 | -42.03639 |
| Brazil | Mato Grosso do Sul | Ceratocystis fimbriata | -20.43556 | -54.86833 |
| Brazil | Mato Grosso do Sul | Ceratocystis fimbriata | -20.47250 | -55.79250 |
| Brazil | Mato Grosso do Sul | Ceratocystis fimbriata | -20.44722 | -55.78194 |
| Brazil | Mato Grosso do Sul | Ceratocystis fimbriata | -20.43889 | -55.74250 |
| Brazil | Mato Grosso do Sul | Ceratocystis fimbriata | -20.34167 | -55.70583 |
| Brazil | Mato Grosso do Sul | Ceratocystis fimbriata | -20.31972 | -55.69528 |
| Brazil | Mato Grosso do Sul | Ceratocystis fimbriata | -20.25417 | -56.07556 |

**S1 Table. Continuation**

| **Country** | **Location name/State** | **Species** | **Latitude** | **Longitude** |
| --- | --- | --- | --- | --- |
| Brazil | Mato Grosso do Sul | Ceratocystis fimbriata | -20.60972 | -54.57528 |
| Brazil | São Paulo | Ceratocystis fimbriata | -21.95278 | -51.71639 |
| Brazil | São Paulo | Ceratocystis fimbriata | -21.97417 | -51.64861 |
| Brazil | São Paulo | Ceratocystis fimbriata | -21.85167 | -51.60750 |
| Brazil | São Paulo | Ceratocystis fimbriata | -21.83750 | -51.60056 |
| Brazil | São Paulo | Ceratocystis fimbriata | -21.87278 | -49.90778 |
| Brazil | São Paulo | Ceratocystis fimbriata | -21.54667 | -49.65111 |
| Brazil | São Paulo | Ceratocystis fimbriata | -21.19583 | -48.05806 |
| Brazil | São Paulo | Ceratocystis fimbriata | -21.18222 | -48.55833 |
| Brazil | São Paulo | Ceratocystis fimbriata | -21.15917 | -48.61056 |
| Brazil | São Paulo | Ceratocystis fimbriata | -21.19861 | -48.63000 |
| Brazil | São Paulo | Ceratocystis fimbriata | -20.42222 | -50.08750 |
| Brazil | São Paulo | Ceratocystis fimbriata | -22.58214 | -47.45397 |
| Brazil | São Paulo | Ceratocystis fimbriata | -21.52483 | -48.52556 |
| Brazil | São Paulo | Ceratocystis fimbriata | -21.47406 | -48.60203 |
| Brazil | São Paulo | Ceratocystis fimbriata | -21.31311 | -48.61428 |
| Brazil | São Paulo | Ceratocystis fimbriata | -21.31075 | -48.59269 |
| Brazil | São Paulo | Ceratocystis fimbriata | -21.29861 | -48.59778 |
| Brazil | Pernambuco | Ceratocystis fimbriata | -8.34931 | -35.01714 |
| Brazil | Pernambuco | Ceratocystis fimbriata | -8.31047 | -35.01489 |
| Brazil | Paraíba | Ceratocystis fimbriata | -7.21417 | -34.84028 |
| Brazil | Paraíba | Ceratocystis fimbriata | -7.25661 | -34.83142 |
| Brazil | Alagoas | Ceratocystis fimbriata | -9.27922 | -37.95972 |
| Brazil | Alagoas | Ceratocystis fimbriata | -9.26472 | -37.92942 |
| Brazil | Bahia | Ceratocystis fimbriata | -10.35933 | -40.17172 |
| Brazil | Bahia | Ceratocystis fimbriata | -10.39136 | -40.17986 |
| Brazil | Bahia | Ceratocystis fimbriata | -10.36106 | -40.16442 |
| Brazil | Ceará | Ceratocystis fimbriata | -6.40428 | -38.90722 |
| Brazil | Ceará | Ceratocystis fimbriata | -7.53692 | -39.00742 |
| Brazil | Ceará | Ceratocystis fimbriata | -7.55106 | -39.01897 |
| Brazil | Rio de Janeiro | Ceratocystis fimbriata | -21.63538 | -41.95150 |
| Brazil | Espirito Santo | Ceratocystis fimbriata | -21.10967 | -41.70429 |
| Brazil | Rio de Janeiro | Ceratocystis fimbriata | -21.53705 | -41.81400 |
| Brazil | Espirito Santo | Ceratocystis fimbriata | -21.11475 | -41.70307 |
| Brazil | Espirito Santo | Ceratocystis fimbriata | -21.11579 | -41.69221 |
| Brazil | Rio de Janeiro | Ceratocystis fimbriata | -21.63708 | -41.95625 |
| Brazil | Rio de Janeiro | Ceratocystis fimbriata | -21.54809 | -41.84062 |
| Brazil | Rio de Janeiro | Ceratocystis fimbriata | -21.19413 | -41.90460 |
| Brazil | Rio de Janeiro | Ceratocystis fimbriata | -21.14734 | -42.06401 |
| Brazil | Rio de Janeiro | Ceratocystis fimbriata | -21.55043 | -41.84065 |
| Brazil | Rio de Janeiro | Ceratocystis fimbriata | -21.65077 | -41.87370 |
| Brazil | Rio de Janeiro | Ceratocystis fimbriata | -21.42793 | -42.20227 |

**S1 Table. Continuation**

| **Country** | **Location name/State** | **Species** | **Latitude** | **Longitude** |
| --- | --- | --- | --- | --- |
| Brazil | Rio de Janeiro | Ceratocystis fimbriata | -21.59937 | -41.85688 |
| Pakistan | Multan Region | Ceratocystis fimbriata | 30.270 | 71.250 |
| Pakistan | Multan Region | Ceratocystis fimbriata | 30.263 | 71.506 |
| Pakistan | Multan Region | Ceratocystis fimbriata | 30.266 | 71.494 |
| Pakistan | Multan Region | Ceratocystis fimbriata | 30.255 | 71.513 |
| Pakistan | Punjab | Ceratocystis fimbriata | 31.410 | 73.050 |
| Pakistan | Punjab | Ceratocystis fimbriata | 30.650 | 73.120 |
| Pakistan | Punjab | Ceratocystis fimbriata | 28.390 | 70.320 |
| Pakistan | Punjab | Ceratocystis fimbriata | 29.400 | 71.660 |
| Pakistan | Sindh Province | Ceratocystis fimbriata | 25.430 | 68.540 |
| Pakistan | Sindh Province | Ceratocystis fimbriata | 25.410 | 68.670 |
| Pakistan | Sindh Province | Ceratocystis fimbriata | 25.460 | 68.720 |
| Pakistan | Sindh Province | Ceratocystis fimbriata | 25.370 | 68.350 |
| Pakistan | Multan Region | Ceratocystis fimbriata | 30.268 | 71.495 |
| Pakistan | Multan Region | Ceratocystis fimbriata | 29.880 | 71.290 |
