# Supplementary material for: Mapping Global Potential Risk of Mango Sudden Decline Disease Caused by Ceratocystis fimbriata
Source: PLoS One. 2016 Jul 14;11(7):e0159450. doi: 10.1371/journal.pone.0159450 (PMC4944967; doi:10.1371/journal.pone.0159450)
Supplement: S2 Table — (DOCX) [file pone.0159450.s006.docx]

**S2 Table. Cross-correlation (Pearson correlation coefficient, r) among environmental variables.**

|  | **bio1** | bio2 | bio3 | bio4 | bio5 | bio6 | bio7 | bio8 | bio9 | bio10 | bio11 | bio12 | bio13 | bio14 | bio15 | bio16 | bio17 | bio18 | bio19 |
| --- | --- | --- | --- | --- | --- | --- | --- | --- | --- | --- | --- | --- | --- | --- | --- | --- | --- | --- | --- |
| **bio2** | 0.525 |  |  |  |  |  |  |  |  |  |  |  |  |  |  |  |  |  |  |
| bio3 | 0.838 | 0.391 |  |  |  |  |  |  |  |  |  |  |  |  |  |  |  |  |  |
| bio4 | -0.833 | -0.217 | -0.893 |  |  |  |  |  |  |  |  |  |  |  |  |  |  |  |  |
| bio5 | 0.896 | 0.708 | 0.611 | -0.513 |  |  |  |  |  |  |  |  |  |  |  |  |  |  |  |
| bio6 | 0.968 | 0.361 | 0.888 | -0.936 | 0.765 |  |  |  |  |  |  |  |  |  |  |  |  |  |  |
| bio7 | -0.732 | 0.006 | -0.829 | 0.972 | -0.360 | -0.876 |  |  |  |  |  |  |  |  |  |  |  |  |  |
| bio8 | 0.811 | 0.534 | 0.635 | -0.500 | 0.844 | 0.703 | -0.387 |  |  |  |  |  |  |  |  |  |  |  |  |
| bio9 | 0.938 | 0.444 | 0.808 | -0.860 | 0.792 | 0.948 | -0.782 | 0.607 |  |  |  |  |  |  |  |  |  |  |  |
| bio10 | 0.935 | 0.624 | 0.657 | -0.584 | 0.989 | 0.825 | -0.455 | 0.864 | 0.831 |  |  |  |  |  |  |  |  |  |  |
| bio11 | 0.980 | 0.431 | 0.891 | -0.926 | 0.796 | 0.996 | -0.848 | 0.730 | 0.950 | 0.847 |  |  |  |  |  |  |  |  |  |
| bio12 | 0.374 | -0.244 | 0.564 | -0.551 | 0.115 | 0.482 | -0.613 | 0.248 | 0.370 | 0.194 | 0.450 |  |  |  |  |  |  |  |  |
| **bio13** | 0.452 | -0.103 | 0.580 | -0.566 | 0.228 | 0.524 | -0.588 | 0.367 | 0.409 | 0.298 | 0.508 | 0.896 |  |  |  |  |  |  |  |
| **bio14** | 0.049 | -0.375 | 0.216 | -0.238 | -0.145 | 0.160 | -0.339 | -0.071 | 0.101 | -0.082 | 0.116 | 0.705 | 0.388 |  |  |  |  |  |  |
| **bio15** | 0.370 | 0.515 | 0.285 | -0.194 | 0.429 | 0.273 | -0.074 | 0.449 | 0.263 | 0.412 | 0.320 | -0.172 | 0.140 | -0.518 |  |  |  |  |  |
| bio16 | 0.441 | -0.123 | 0.581 | -0.567 | 0.210 | 0.519 | -0.594 | 0.349 | 0.404 | 0.281 | 0.501 | 0.922 | 0.993 | 0.425 | 0.095 |  |  |  |  |
| bio17 | 0.076 | -0.372 | 0.251 | -0.269 | -0.126 | 0.190 | -0.370 | -0.049 | 0.128 | -0.060 | 0.146 | 0.741 | 0.425 | 0.994 | -0.516 | 0.463 |  |  |  |
| bio18 | 0.216 | -0.203 | 0.348 | -0.332 | 0.019 | 0.275 | -0.385 | 0.249 | 0.145 | 0.093 | 0.259 | 0.795 | 0.742 | 0.552 | -0.103 | 0.761 | 0.576 |  |  |
| **bio19** | 0.245 | -0.241 | 0.429 | -0.401 | 0.053 | 0.353 | -0.472 | 0.071 | 0.304 | 0.109 | 0.313 | 0.751 | 0.581 | 0.668 | -0.273 | 0.607 | 0.696 | 0.369 |  |
| **Elevation** | -0.189 | 0.172 | 0.017 | -0.049 | -0.294 | -0.143 | -0.013 | -0.340 | -0.101 | -0.322 | -0.121 | -0.085 | -0.064 | -0.068 | 0.149 | -0.064 | -0.070 | -0.017 | -0.089 |

Note: Bold font indicates variables in the final model. Only one variable from a group of highly correlated variables was included in the models (Pearson correlation coefficient, r ≥ |0.70|).
